# Triglyceride glucose-body mass index is associated with diabetic kidney disease in type 2 diabetes mellitus patients without non-alcoholic fatty liver disease

**DOI:** 10.3389/fnut.2025.1628867

**Published:** 2025-07-16

**Authors:** Yafen Chu, Hongping Yao, Ni Lu, Yu Chang, Jumei Wang, Linlin Xu, Bing Song, Songtao Feng, Hui Zhang

**Affiliations:** ^1^Department of Endocrinology, Ningbo Medical Center Lihuili Hospital, Ningbo, China; ^2^Department of Endocrinology, The First Affiliated Hospital of USTC, Division of Life Sciences and Medicine, University of Science and Technology of China, Hefei, China; ^3^Department of Endocrinology, The First Affiliated Hospital of Jinzhou Medical University, Jinzhou, China; ^4^Department of Nephrology, Jiangsu University Affiliated People’s Hospital, Zhenjiang, China; ^5^Endocrinology and Metabolism Center, The First Affiliated Hospital, and College of Clinical Medicine of Henan University of Science and Technology, Luoyang, China

**Keywords:** triglyceride glucose-body mass index, diabetic kidney disease, diabetic complication, non-alcoholic fatty liver disease, type 2 diabetes mellitus

## Abstract

**Aim:**

The triglyceride glucose-body mass index (TyG-BMI) is correlated not only to the onset of diabetic kidney disease (DKD) associated with nutrition (including glucose and triglyceride) metabolism but also incorporates body mass index (BMI), potentially serving as an indicator of non-alcoholic fatty liver disease (NAFLD). But, the links between TyG-BMI and DKD in individuals with and without NAFLD remains further exploration. Therefore, this present research aims to investigate the relationship between TyG-BMI and DKD in these patient groups and to evaluate its diagnostic value for DKD.

**Methods:**

A total of 425 individuals diagnosed with type 2 diabetes mellitus (T2DM) were categorized into the DKD group or the Control group. Clinical indicators were compared in these two groups, and the relationships between TyG-BMI and either the estimated glomerular filtration rate (eGFR) or the urinary albumin-to-creatinine ratio (UACR) were examined. Risk factors for DKD were examined, and the diagnostic value of TyG-BMI was assessed in patients without and with NAFLD.

**Results:**

Compared to 288 participants without DKD, 137 individuals with DKD exhibited higher TyG-BMI levels. In all individuals with T2DM, a higher TyG-BMI was recognized as a contributing factor to the development of DKD. Notably, this association was observed in patients without NAFLD, while no such link was established in those diagnosed with NAFLD. For T2DM patients lacking NAFLD, the TyG-BMI threshold of 49.2415 demonstrated a sensitivity of 91.3% and a specificity of 70.1%.

**Conclusion:**

Increased TyG-BMI values are associated with a higher risk of DKD and could act as potential indicators of DKD in individuals with T2DM.

## 1 Introduction

Type 2 diabetes mellitus (T2DM) is a long-term metabolic disease marked by impaired insulin sensitivity and dysfunction of pancreatic β-cells (1). It is not only associated with significant disturbances in glucose metabolism but is also frequently accompanied by lipid metabolism abnormalities (2, 3). The global incidence of T2DM is steadily increasing. As reported by the International Diabetes Federation in 2021, over 530 million individuals around the world are currently affected by diabetes, and more than 90% of these cases are attributed to T2DM. China, ranking among the nations with the highest rates of diabetes, has an adult diabetes prevalence of around 12.8% for individuals aged 18 and above, according to data from national surveillance on chronic diseases and nutrition. This represents a significant public health burden (4). With the increasing prevalence of diabetes, diabetic kidney disease (DKD) has become one of the primary contributors to end-stage renal disease (ESRD) (5, 6). The pathogenesis of DKD involves multiple factors, including disturbances in nutritional metabolism—particularly insulin resistance associated abnormalities in glucose and lipid metabolism—renal fibrosis, oxidative stress, microcirculatory dysfunction, and chronic metabolic inflammation (7). Among these, disruptions in glucose (8–10) and lipid (11, 12) metabolism play a central initiating role. These metabolic disturbances trigger a cascade of pathological mechanisms that ultimately result in reduced estimated glomerular filtration rate (eGFR) and an increase in urinary protein excretion. As proteinuria worsens and renal function declines, patients with diabetes are placed on a fast track toward ESRD. Therefore, early identification, timely diagnosis, and intervention for DKD are essential strategies to prevent progression to ESRD in diabetic patients.

Currently, the diagnosis of DKD primarily relies on the urinary albumin-to-creatinine ratio (UACR) and eGFR, along with the exclusion of other causes of proteinuria and renal function decline. In some cases, pathological examination is required to confirm the diagnosis (13). However, the current diagnostic methods are relatively complex. While pathological assessment can provide a definitive diagnosis, its invasive nature often makes it unacceptable to many patients. Moreover, using UACR and eGFR alone does not directly account for abnormalities in glucose and lipid metabolism, necessitating the exclusion of other renal diseases. Therefore, it is imperative to discover novel biomarkers that can help screen high-risk individuals and enable a more efficient diagnosis of DKD.

The triglyceride glucose (TyG) index serves as an integrated indicator, reflects both glucose and lipid metabolism disorders. In recent years, it has shown significant advantages in the diagnosis of chronic complications of diabetes (14–16). Not only can it aid in diagnosing these conditions, but it may also help predict disease prognosis. Although some studies have indicated a possible link between chronic kidney disease (CKD) and the TyG index in diabetic individuals (17), no research to date has evaluated its diagnostic value for CKD specifically. Earlier researches have established a strong association between obesity and both diabetes (18) and its related complications (19), such as DKD (20). Body mass index (BMI) is one of the indicators reflecting the degree of obesity in patients. Therefore, taking body mass index into account, TyG-BMI may be more closely associated with diabetic complications. Some researchers have also proposed that the triglyceride glucose-body mass index (TyG-BMI) might have a strong correlation with diabetic complications (21–25), including DKD (26).

Based on the above hypothesis, this study preliminarily explored the relationship between the TyG and TyG-BMI with DKD, and further evaluated their potential diagnostic value for CKD. Disorders of glucose and lipid metabolism not only linked to the initiation and advancement of DKD but are also closely associated with the development of non-alcoholic fatty liver disease (NAFLD). While DKD and NAFLD share several pathogenic mechanisms, they also differ in many aspects of their pathophysiology. To minimize potential confounding factors, we additionally examined the relationship of the TyG index and TyG-BMI with DKD in patients both with and without coexisting NAFLD.

## 2 Materials and methods

### 2.1 Study design and ethical approval

A total of 425 individuals diagnosed with T2DM were included from the Department of Endocrinology, the First Affiliated Hospital of USTC. Of these, 137 patients with DKD were placed in the DKD group, whereas the other 288 patients without DKD served as the control group. According to the Asia-Pacific guidelines (27), the diagnosis of NAFLD was established when at least two out of the following three sonographic features were present on abdominal ultrasound: increased hepatic echogenicity compared to the kidney or spleen, poor visualization of intrahepatic vessels, and attenuation of the ultrasound beam in deeper liver regions. The diagnosis was strongly suspected after excluding other possible causes of liver dysfunction—particularly significant consumption of alcohol (exceeding 140 g per week for males and 70 g for females) and the administration of liver-damaging drugs—had been carefully excluded. Prior to enrollment, every participant received comprehensive information regarding the study’s objectives and procedures, and gave their written informed consent. Ethical approval for the study was approved by the Ethics Committee of the First Affiliated Hospital of USTC (Approval No.: 2025-RE-045).

### 2.2 Inclusion and exclusion criteria

Every individual involved in the research was identified as having diabetes according to the 1999 guidelines established by the World Health Organization (28). Clinicians recorded the diagnosis of DKD in the medical history system according to the guideline of American Diabetes Association (29). Exclusion criteria were defined for this study focusing on NAFLD and DKD: (a) types of diabetes other than T2DM; (b) presence of acute diabetic complications (such as lactic acidosis, hyperglycemic hyperosmolar state, or diabetic ketoacidosis); (c) history of severe hypoglycemia; (d) severe cardiovascular or cerebrovascular diseases; (e) other vascular disorders; (f) venous embolism or lymphangitis; (g) history of any amputation; (h) thyroid dysfunction; (i) smoking; (j) alcohol consumption exceeding 140 g per week for males and 70 g for females; (k) presence of other liver diseases, including viral hepatitis infections, biliary obstruction diseases, or autoimmune hepatitis; and (l) use of medications known to affect liver function, such as tamoxifen, amiodarone, sodium valproate, methotrexate, or glucocorticoids.

### 2.3 Clinical data

Patient clinical information was gathered, encompassing factors such as age, gender, and the duration of diabetes. At the time of admission, measurements including height and weight were recorded, and the body mass index (BMI) was determined using the formula: weight in kilograms divided by the square of height in meters (kg/m^2^). On the second day after admission, blood samples were collected to evaluate the parameters of glucose metabolism, including glycated hemoglobin (HbA1c), fasting plasma glucose (FPG), and fasting serum C-peptide (FCP); lipid metabolism, such as triglycerides (TG), total cholesterol (TC), low-density lipoprotein cholesterol (LDL-C), and high-density lipoprotein cholesterol (HDL-C); renal function markers, including serum uric acid (SUA), creatinine (Cr), and blood urea nitrogen (BUN); as well as liver function indicators, alanine aminotransferase (ALT), and aspartate aminotransferase (AST). All laboratory tests were conducted at the Central Laboratory of the First Affiliated Hospital of USTC. The eGFR was estimated based on serum creatinine concentrations. Furthermore, urine specimens were gathered to assess microalbuminuria and urinary creatinine levels, which enabled the determination of the UACR. All data were obtained from the medical records of the patients.

### 2.4 Statistical methods

Statistical analysis was conducted utilizing SPSS version 22.0 (IBM, USA). Variables exhibiting a normal distribution, including LDL-C and TyG-BMI, were reported as means ± standard deviations, and comparisons between groups were made by the Student’s *t*-test. For variables that did not follow a normal distribution—such as age, duration of diabetes, BMI, HbA1c, FPG, FCP, TG, TC, HDL-C, TyG, SUA, Cr, BUN, eGFR, ALT, AST, urinary albumin, and UACR—data were presented as medians and interquartile ranges. Their differences of the two groups were analyzed via the Mann–Whitney *U* test. Categorical data, including gender and NAFLD status, were expressed as counts and percentages. Their differences of the two groups were evaluated by the Chi-squared test. To investigate correlations, Pearson and partial correlation analyses were performed both unadjusted and adjusted for covariates. Furthermore, binary logistic regression analysis was employed to identify potential risk factors for DKD. Receiver operating characteristic (ROC) curve analysis was also carried out to establish optimal threshold values and evaluate diagnostic accuracy among all patients, regardless of NAFLD presence. Youden index was used to establish the diagnostic cut-off point. The methodology section encompasses the study design and ethical approval, data collection and statistical analyses were conducted in accordance with our previously published studies (30).

## 3 Results

### 3.1 Analysis of clinical features among T2DM individuals with or without DKD

This research initially examined the clinical profiles of individuals with T2DM, both with and without DKD. Despite the cross-sectional design and lack of strict matching between the two cohorts, no notable disparities were observed regarding sex or the duration of diabetes mellitus (DDM). Additionally, factors such as BMI, FCP, TC, LDL-C, AST, and ALT levels were similarly distributed between the groups (all *P* > 0.05). However, patients with DKD were older compared to those without DKD (*P* = 0.049). HbA1c, FPG, and TG, as well as lower HDL-C concentrations were elevated in T2DM individuals with DKD relative to those without the condition (all *P* < 0.05). Given the presence of glucose and lipid metabolism abnormalities in this research, we additionally computed the TyG index and TyG-BMI for more detailed analysis. This study aimed to investigate the potential role of TyG (TyG-BMI) in T2DM patients with DKD. To achieve this, we compared TyG (TyG-BMI) levels and renal function markers—including SUA, Cr, BUN, eGFR, urinary albumin, and UACR—between patients with and without DKD. Our results demonstrated that individuals with DKD had significantly elevated TyG and TyG-BMI, compared to those without DKD (all *P* < 0.05). Moreover, individuals with DKD exhibited decreased eGFR and elevated Cr, urinary albumin, and UACR levels compared to their counterparts without DKD (all *P* < 0.05) (see Table 1).

**TABLE 1:** Comparison of clinical parameters between Control group and DKD group.

	Control (*n* = 288)	DKD (*n* = 137)	*P*
Age (year)	61.00 (56.00, 70.00)	65.00 (58.00, 72.00)	0.049^a^ *
Female (*n*, %)	122, 42.36	59, 43.07	0.891^c^
DDM (year)	10.00 (4.00, 17.00)	10.00 (8.00, 20.00)	0.149^a^
BMI (kg/m^2^)	23.66 (21.55, 26.02)	24.10 (22.50, 27.13)	0.388^a^
HbA1c (%)	7.70 (6.80, 9.10)	8.40 (7.15, 9.60)	0.001^a^ *
FPG (mmol/L)	6.80 (5.48, 8.28)	7.88 (5.97, 9.89)	0.017^a^ *
FCP (nmol/L)	0.48 (0.32, 0.66)	0.55 (0.36, 0.80)	0.092^a^
TG (mmol/L)	1.23 (0.96, 1.83)	1.67 (1.32, 2.21)	<0.001^a^ *
TC (mmol/L)	4.28 (3.60, 4.92)	4.26 (3.54, 4.91)	0.973^a^
HDL-C (mmol/L)	1.03 (0.88, 1.23)	0.98 (0.83, 1.15)	0.047^a^ *
LDL-C (mmol/L)	2.51 ± 0.80	2.57 ± 0.87	0.442^b^
TyG	2.18 (1.79, 2.60)	2.55 (2.22, 2.98)	<0.001^a^ *
TyG-BMI	55.03 ± 20.80	63.88 ± 13.61	<0.001^b^ *
NAFLD (*n*, %)	154, 53.47	68, 49.64	0.459^c^
AST (U/L)	19.00 (14.00, 25.88)	17.00 (13.15, 24.50)	0.426^a^
ALT (U/L)	18.60 (16.00, 22.00)	19.00 (16.00, 24.00)	0.758^a^
SUA (μmol/L)	304.00 (248.19, 353.00)	323.00 (268.50, 381.50)	0.090^a^
Cr (μmol/L)	59.50 (50.25, 70.00)	66.00 (53.00, 92.00)	0.012^a^ *
BUN (mmol/L)	6.27 (5.23, 7.40)	6.91 (5.55, 8.03)	0.137^a^
eGFR (ml/min/1.73 m^2^)	97.21 (90.31, 105.11)	90.23 (71.55, 100.72)	<0.001^a^ *
Urinary albumin (mg/L)	7.65 (4.80, 12.18)	51.40 (26.60, 229.90)	<0.001^a^ *
UACR (mg/g)	8.85 (5.98, 13.95)	97.15 (39.55, 323.23)	<0.001^a^ *

DKD, diabetic kidney disease; DDM, duration of diabetes mellitus; BMI, body mass index; HbA1c, glycosylated hemoglobin; FPG, fasting plasma glucose; FCP, fasting C-peptide; TG, triglycerides; TC, total cholesterol; LDL-C, low-density lipoprotein cholesterol; HDL-C, high-density lipoprotein cholesterol; TyG, triglyceride glucose index; TyG-BMI, triglyceride glucose-body mass index; NAFLD, non-alcoholic fatty liver disease; ALT, alanine aminotransferase; AST, aspartate aminotransferase; SUA, serum uric acid; Cr, creatinine; BUN, blood urea nitrogen; eGFR, estimated glomerular filtration rate; UACR, urinary albumin-to-creatinine ratio.

*^a^*The Mann–Whitney *U* test was employed for asymmetrically distributed variables.

*^b^*Student’s *t*-test was employed for normally distributed variables.

*^c^*The Chi-square test was employed for categorical variables.

^*^P < 0.05.

### 3.2 Correlation of TyG index (TyG-BMI) with renal function in individuals diagnosed with T2DM

To explore the possible relationship between TyG (TyG-BMI) and renal function among patients with T2DM, Pearson’s correlation analysis was conducted. Among individuals with T2DM, a notable association was identified between UACR and both TyG (*R* = 0.128, *P* = 0.008) as well as TyG-BMI (*R* = 0.121, *P* = 0.012), indicating statistically significant positive relationships. Additionally, partial correlation analyses were conducted with adjustments for age, sex, and DDM. After these modifications, an inverse association was observed between TyG values and eGFR (*R* = −0.098, *P* = 0.044), while a direct relationship was identified with UACR (*R* = 0.139, *P* = 0.004). In a comparable manner, TyG-BMI was inversely related to eGFR (*R* = −0.106, *P* = 0.030) and positively linked to UACR (*R* = 0.130, *P* = 0.008) (see Table 2).

**TABLE 2:** Association between TyG-BMI (or TyG) and UACR (or eGFR) in patients with T2DM.

		TyG		TyG-BMI	
		*R*	*P*	*R*	*P*
Model 1	eGFR	−0.013	0.784	−0.038	0.430
	UACR	0.128	0.008*	0.121	0.012*
Model 2	eGFR	−0.098	0.044*	−0.106	0.030*
	UACR	0.139	0.004*	0.130	0.008*

Model 1 showed the Pearson association between TyG-BMI (or TyG) and UACR (or eGFR) in patients with T2DM; model 2 showed the partial association between TyG-BMI (or TyG) and UACR (or eGFR) in patients with T2DM adjusting for age and gender as well as duration of diabetes mellitus. TyG-BMI, triglyceride glucose-body mass index; TyG, triglyceride glucose index; T2DM, type 2 diabetes mellitus; eGFR, estimated glomerular filtration rate; UACR, urinary albumin-to-creatinine ratio.

*P < 0.05.

### 3.3 Analysis for the risk factor for DKD in patients with T2DM

Given that TyG and TyG-BMI are associated with eGFR and UACR—both markers of kidney function—we hypothesized that TyG and TyG-BMI could act as possible contributors to the development of DKD in patients diagnosed with T2DM. To explore this possibility, we performed a binary logistic regression analysis. Our results showed that higher TyG and TyG-BMI levels were significantly associated with an increased risk of DKD in patients with T2DM (OR = 2.225, *P* < 0.001; OR = 1.025, *P* < 0.001) (see Supplementary Table 1). Moreover, even after adjusting for age, gender, and DDM, elevated TyG and TyG-BMI still represented notable risk contributors to DKD in this population (OR = 2.499, *P* < 0.001; OR = 1.028, *P* < 0.001) (see Supplementary Table 2).

### 3.4 Difference of the relationship between TyG (TyG-BMI) and DKD in T2DM patients with and without NAFLD

Because TyG and TyG-BMI may be linked not only to DKD but also closely to NAFLD, we performed separate analyses to examine the relationships between TyG (TyG-BMI) and DKD among patients both with and without NAFLD, aiming to minimize potential confounding effects. Among patients without NAFLD, TyG (TyG-BMI) was significantly correlated with both eGFR and UACR, before and after adjusting for gender, sex, and DDM (all *P* < 0.05). In contrast, among T2DM patients with NAFLD, no meaningful correlations were identified involving TyG (or TyG-BMI) and either eGFR or UACR, regardless of adjustment (all *P* > 0.05) (Table 3). These findings point to a complex interplay among TyG (TyG-BMI), DKD, and NAFLD. Given this complexity, we investigate the association between TyG (TyG-BMI) and DKD, and performed supplementary subgroup evaluations employing binary logistic regression. Subgroup evaluation demonstrated that among T2DM individuals lacking NAFLD, elevated TyG (TyG-BMI) values were independently linked to a greater likelihood of developing DKD, irrespective of adjustment for age, gender, and DDM (all *P* < 0.05). However, this association was not observed in diabetic patients with NAFLD (all *P* > 0.05) (see Supplementary Tables 3, 4).

**TABLE 3:** Association between TyG-BMI (or TyG) and UACR (or eGFR) in T2DM patients with and without NAFLD.

			Patients without NAFLD		Patients with NAFLD	
			*R*	*P*	*R*	*P*
TyG	Model 1	eGFR	−0.141	0.045*	0.077	0.254
		UACR	0.260	<0.001*	−0.015	0.822
	Model 2	eGFR	−0.188	0.008*	−0.020	0.774
		UACR	0.259	<0.001*	0.041	0.550
TyG-BMI	Model 1	eGFR	0.215	0.002*	0.062	0.356
		UACR	0.290	<0.001*	0.014	0.832
	Model 2	eGFR	−0.270	<0.001*	0.018	0.778
		UACR	0.289	<0.001*	0.052	0.447

Model 1 showed the Pearson association between TyG-BMI (or TyG) and UACR (or eGFR) in patients with T2DM; model 2 showed the partial association between TyG-BMI (or TyG) and UACR (or eGFR) in patients with T2DM adjusting for age and gender as well as duration of diabetes mellitus. TyG-BMI, triglyceride glucose-body mass index; TyG, triglyceride glucose index; eGFR, estimated glomerular filtration rate; UACR, urinary albumin-to-creatinine ratio; NAFLD, non-alcoholic fatty liver disease; T2DM, type 2 diabetes mellitus.

*P < 0.05.

### 3.5 Assessing the diagnostic utility of TyG (TyG-BMI) in detecting DKD among individuals with T2DM

**FIGURE 1:**
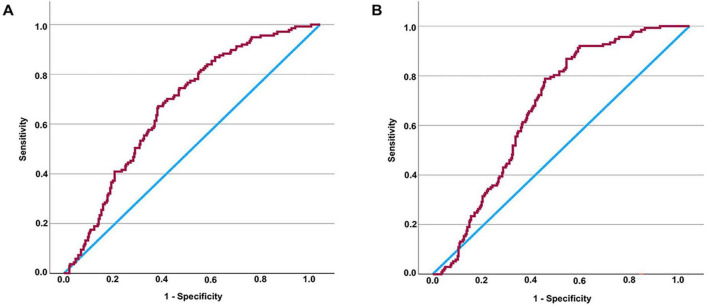
Assessing the diagnostic values of TyG (or TyG-BMI) for DKD in patients with T2DM by ROC curve. AUC of TyG in all patients = 0.674 (*P* < 0.001) **(A)**. It is determined that the diagnostic cut-off value of TyG for DKD in all patients with T2DM to be 2.3789, revealed a sensitivity of 67.2% and specificity of 63.2% (Youden index = 0.304). AUC of TyG-BMI in all patients = 0.673 (*P* < 0.001) **(B)**. It is determined that the diagnostic cut-off value of TyG-BMI for DKD in all patients with T2DM to be 53.8295, revealed a sensitivity of 78.8% and specificity of 56.2% (Youden index = 0.350). TyG-BMI, triglyceride glucose-body mass index; TyG, triglyceride glucose index; DKD, diabetic kidney disease; T2DM, type 2 diabetes mellitus; ROC, receiver operating characteristic.

**FIGURE 2:**
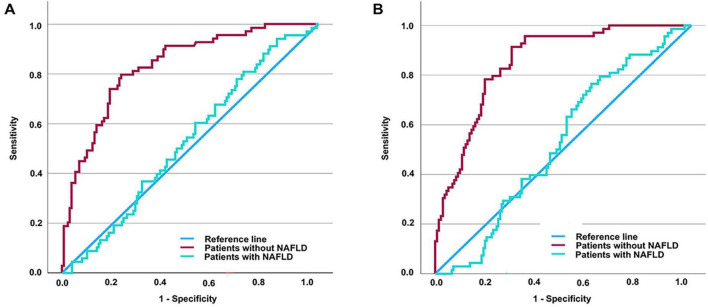
Assessing the diagnostic values of TyG (or TyG-BMI) for DKD in T2DM patients with and without NAFLD by ROC curve. AUC of TyG in patients without NAFLD = 0.833 (*P* < 0.001); AUC of TyG in patients with NAFLD = 0.529 (*P* = 0.478) **(A)**. AUC of TyG-BMI in patients without NAFLD = 0.854 (*P* < 0.001); AUC of TyG-BMI in patients with NAFLD = 0.528 (*P* = 0.473) **(B)**. It is determined that the diagnostic cut-off value of TyG for DKD in T2DM without NAFLD to be 2.316, revealed a sensitivity of 79.7% and specificity of 76.9% (Youden index = 0.566). It is determined that the diagnostic cut-off value of TyG-BMI for DKD in T2DM without NAFLD to be 49.2415, revealed a sensitivity of 91.3% and specificity of 70.1% (Youden index = 0.614). TyG-BMI, triglyceride glucose-body mass index; TyG, triglyceride glucose index; DKD, diabetic kidney disease; T2DM, type 2 diabetes mellitus; NAFLD, non-alcoholic fatty liver disease; ROC, receiver operating characteristic.

Since increased TyG and TyG-BMI values are recognized as possible indicators of DKD risk in individuals with T2DM, we performed an extensive assessment of their ability to diagnose the condition throughout the full T2DM population. ROC curve assessment showed that the area under the curve (AUC) was 0.674 for TyG and 0.673 for TyG-BMI in all T2DM patients (Figures 1A, B). Building on our previous subgroup analyses, we further assessed the diagnostic significance of TyG and TyG-BMI specifically among T2DM patients both diagnosed with and without NAFLD. The optimal cut-off values were determined to be 2.316 for TyG and 49.2415 for TyG-BMI. For TyG, the analysis yielded a sensitivity of 79.7%, a specificity of 76.9%, and an AUC of 0.833 (Youden index = 0.566). For TyG-BMI, a sensitivity of 91.3%, a specificity of 70.1%, and an AUC of 0.854 were observed (Youden index = 0.614) (Figure 2B). In contrast, among patients diagnosed with NAFLD, the diagnostic performance of TyG and TyG-BMI was substantially lower, with AUCs of only 0.529 and 0.528, respectively (Figures 2A, B).

## 4 Discussion

As diabetes becomes increasingly widespread, DKD has rapidly become a pressing issue for public health authorities. As outlined in the introduction, DKD has become a major contributor to ESRD (6). In China, its incidence is particularly high, placing a significant burden on society (31). Consequently, early detection and timely intervention for DKD are especially critical. However, diagnosing DKD remains relatively complex, requiring the exclusion of many other potential causes of kidney injury. Regarding the pathogenesis of DKD, Disruptions in glucose and lipid metabolism associated with reduced insulin sensitivity are critically important. Therefore, in this research, we aimed to investigate the association between DKD and the TyG index among individuals diagnosed with T2DM, given that the TyG index reflects both glucose and lipid metabolism and is closely linked to insulin resistance. Considering that DKD is associated not only with persistent hyperglycemia but also with obesity, we also explored how the TyG-BMI measure correlates with and DKD, in addition to the TyG index alone. Our preliminary findings revealed that individuals in the DKD group exhibited markedly elevated values of TyG and TyG-BMI relative to those in the Control group.

Since there were differences in TyG and TyG-BMI levels between T2DM individuals with DKD and those without, we further explored how these markers correlate with kidney function. Pearson correlation analyses indicated a significant relationship between UACR and both the TyG index as well as TyG-BMI. Although this was a cross-sectional study without strict matching for age, sex, and diabetes duration, there were no notable variations between the two groups in terms of gender or the length of time since diabetes diagnosis. Although individuals with DKD tended to be older compared to those without the condition, the difference in age was only marginally significant (*P* = 0.049). Despite the minimal differences in age, gender, and DDM between the groups, we still adjusted for these factors in subsequent correlation analyses. Interestingly, after adjusting for age, gender, and DDM, partial correlation analyses showed an inverse association between TyG (or TyG-BMI) and eGFR, while a direct relationship was observed with UACR—two key indicators in the diagnosis of DKD (29). These findings indicate a significant link between TyG (or TyG-BMI) and DKD. To further clarify whether TyG and TyG-BMI serve as independent predictors of DKD among individuals with T2DM, we conducted regression analyses. The results clearly demonstrated that both TyG and TyG-BMI were independent predictors of DKD, regardless of adjustments for age, sex, and diabetes duration. Finally, ROC curve analysis was conducted to evaluate the potential of TyG and TyG-BMI in identifying DKD. The AUC values were 0.674 and 0.673, respectively, indicating that their diagnostic performance in the overall population might be limited.

One of the most frequently occurring coexisting conditions among individuals with T2DM is NAFLD (32). Its development is closely associated not only with insulin resistance but also with lipid metabolism disorders (33). NAFLD plays a role in initiating and advancing diabetes, and it could elevate the likelihood of diabetes-related complications (34, 35). Several researches have indicated that TyG index is correlated not only with DKD (17) but also with NAFLD (36). Therefore, in our further research, we conducted additional analyses to investigate how TyG, TyG-BMI, and DKD are associated in individuals diagnosed with or without NAFLD. We discovered notable correlations between TyG, TyG-BMI, and renal function markers such as eGFR and UACR among individuals free from NAFLD, and they emerged as independent risk factors for the development of DKD in T2DM patients. However, these associations were not observed in patients with NAFLD. Indeed, in individuals diagnosed with NAFLD, the ROC curve evaluation showed that the diagnostic accuracy of TyG and TyG-BMI for DKD was limited, with respective AUC values of just 0.529 and 0.528. In contrast, among patients without NAFLD, the AUCs for TyG and TyG-BMI were as high as 0.833 and 0.854, respectively. The findings indicate that both TyG and TyG-BMI possess significant potential in diagnosing DKD in individuals free from NAFLD, with TyG-BMI possibly demonstrating a better diagnostic capability. Specifically, when using 49.2415 as the cutoff value for TyG-BMI in diagnosing DKD, the sensitivity and specificity reached 91.3% and 70.1%, respectively. These results suggest that TyG-BMI could function as a valuable initial screening indicator for DKD in T2DM patients who do not have NAFLD. Nevertheless, additional research involving a greater number of participants is required to confirm these conclusions.

To the best of our knowledge, this is the first study to explore the relationship between TyG-BMI and DKD in populations with and without NAFLD. While our study yielded several interesting findings, it also has certain limitations. First, we did not observe a significant difference in the prevalence of NAFLD between T2DM patients with and without DKD. However, recognizing that NAFLD may serve as a potential confounding factor in the mechanistic link between TyG-BMI and DKD, we conducted additional statistical analyses. Admittedly, had we found a significant baseline difference in NAFLD prevalence between the DKD and non-DKD groups, such a result would have provided stronger support for our hypothesis. Nonetheless, in the spirit of scientific rigor, we reported the results as observed. We speculate that the lack of a significant difference may be due to the relatively small sample size. Although our study included over 400 participants, this number is still modest compared to large-scale studies. We acknowledge this as a limitation of our research. Moreover, our study is cross-sectional research, which enables the identification of associations but not causal relationships. This is another inherent limitation that must be considered when interpreting our findings. Additionally, our dataset lacked detailed information on medication use. While we did collect data on whether participants were taking certain medications—such as antidiabetic, antihypertensive, lipid-lowering, and anticoagulant drugs, especially antiplatelet agents—the limited sample size meant that some medications were used by only a few individuals, restricting our ability to analyze their potential impact on DKD. Furthermore, we recorded only the presence or absence of medication use, without details on dosage or duration, which further limited our analysis. Another limitation is the lack of coagulation function data. DKD is a microvascular complication of diabetes, and in a separate study, we found that coagulation markers—particularly activated partial thromboplastin time—are associated with diabetic peripheral neuropathy (37), itself linked to microvascular dysfunction. However, because coagulation data were missing for most participants in this study, we could not investigate this potential relationship here. Alcohol consumption is another factor that can influence diabetic complications (38). To accurately diagnose NAFLD, we excluded patients with excessive alcohol intake at baseline. However, beyond this exclusion, we did not collect detailed information on alcohol consumption. Similarly, because of the known impact of smoking (including second hand smoking) on diabetic complications (39, 40), we excluded smokers at the outset. Still, more granular data on smoking status and intensity would have allowed us to further explore the association between smoking and DKD. Since these were not central to our research question, such data were not incorporated into the study design. It is also important to note that passive smoking may contribute to diabetes-related complications and should be considered in future studies focused on smoking-related risks. In the absence of these data, we recognize this as another limitation of our study.

## 5 Conclusion

In summary, this research reveals that elevated TyG and TyG-BMI values are independently linked to an increased likelihood of DKD among patients with T2DM. Notably, the relationship of TyG and TyG-BMI with DKD varies significantly based on whether NAFLD is present or not. For individuals with T2DM without NAFLD, TyG and TyG-BMI could function as promising indicators for the initial identification of DKD. Moreover, TyG-BMI appears to have greater diagnostic value compared to TyG.

## Data Availability

The raw data supporting the conclusions of this article will be made available by the authors, without undue reservation.
