# Supplementary material for: Triglyceride glucose-body mass index is associated with diabetic kidney disease in type 2 diabetes mellitus patients without non-alcoholic fatty liver disease
Source: Front Nutr. 2025 Jul 16;12:1628867. doi: 10.3389/fnut.2025.1628867 (PMC12307136; doi:10.3389/fnut.2025.1628867)
Supplement: Supplementary file 1 [file Table_1.docx]

Supplementary-Table 1: Assessment of risk factors for DKD in patients with T2DM by binary logistic analysis

|  | β | P | OR | 95% CI | |
| --- | --- | --- | --- | --- | --- |
|  |  |  |  | Lower | Upper |
| TyG | 0.800 | <0.001^*^ | 2.225 | 1.611 | 3.074 |
| TyG-BMI | 0.024 | <0.001^*^ | 1.025 | 1.013 | 1.036 |

Notes: ^*^ P<0.05. Abbreviations: DKD, diabetic kidney disease; T2DM, type 2 diabetes mellitus; triglyceride-glucose body mass index, TyG-BMI; triglyceride glucose index, TyG.

Supplementary-Table 2: Assessment of risk factors for DKD in patients with T2DM by multiple linear regression analysis

|  | β | P | OR | 95% CI | |
| --- | --- | --- | --- | --- | --- |
|  |  |  |  | Lower | Upper |
| TyG | 0.916 | <0.001^*^ | 2.499 | 1.786 | 3.496 |
| Age | 0.047 | <0.001^*^ | 1.048 | 1.020 | 1.077 |
| Gender | 0.075 | 0.737 | 1078 | 0.696 | 1.669 |
| DDM | 0.013 | 0.213 | 1.013 | 0.993 | 1.033 |
| TyG-BMI | 0.027 | <0.001^*^ | 1.028 | 1.016 | 1.039 |
| Age | 0.042 | 0.002^*^ | 1.043 | 1.015 | 1.070 |
| Gender | 0.075 | 0.735 | 1.078 | 0.699 | 1.662 |
| DDM | 0.014 | 0.175 | 1.014 | 0.994 | 1.035 |

Notes: ^*^ P<0.05. Abbreviations: DKD, diabetic kidney disease; T2DM, type 2 diabetes mellitus; triglyceride glucose index, TyG; DDM, duration of diabetes mellitus; triglyceride-glucose body mass index, TyG-BMI.

Supplementary-Table 3: Assessment of risk factors for DKD by binary logistic analysis in T2DM patients with and without NAFLD

|  | Patients without NAFLD | | | | | | Patients without NAFLD | | | | |
| --- | --- | --- | --- | --- | --- | --- | --- | --- | --- | --- | --- |
|  | β | P | | OR | 95% CI | | β | P | OR | 95% C | |
|  |  |  |  |  | Lower | Upper |  |  |  | Lower | Upper |
| TyG | 2.551 | <0.001^*^ | 12.817 | | 5.888 | 27.901 | 0.066 | 0.761 | 1.068 | 0.700 | 1.630 |
| TyG-BMI | 0.113 | <0.001^*^ | 1.120 | | 1.083 | 1.157 | 0.000 | 0.968 | 1.000 | 0.985 | 1.015 |

Notes: ^*^ P<0.05. Abbreviations: DKD, diabetic kidney disease; T2DM, type 2 diabetes mellitus; NAFLD, nonalcoholic fatty liver disease; triglyceride-glucose body mass index, TyG-BMI; triglyceride glucose index, TyG.

Supplementary-Table 4: Assessment of risk factors for DKD by multiple linear regression analysis in T2DM patients with and without NAFLD

|  | Patients without NAFLD | | | | | | Patients without NAFLD | | | | | |
| --- | --- | --- | --- | --- | --- | --- | --- | --- | --- | --- | --- | --- |
|  | β | P | | OR | 95% CI | | β | P | | OR | 95% C | |
|  |  |  |  |  | Lower | Upper |  |  |  |  | Lower | Upper |
| TyG | 2.615 | <0.001^*^ | 13.673 | | 6.150 | 30.398 | 0.189 | | 0.408 | 1.208 | 0.772 | 1.891 |
| Age | 0.029 | 0.193 | 1.029 | | 0.986 | 1.074 | 0.059 | | 0.002^*^ | 1.061 | 1.023 | 1.101 |
| Gender | 0.140 | 0.703 | 1.150 | | 0.561 | 2.359 | -0.138 | | 0.655 | 0.871 | 0.476 | 1.595 |
| DDM | 0.022 | 0.345 | 1.022 | | 0.977 | 1.068 | 0.008 | | 0.425 | 1.008 | 0.988 | 1.029 |
| TyG-BMI | 0.118 | <0.001^*^ | 1.125 | | 1.087 | 1.165 | 0.002 | | 0.837 | 1.002 | 0.986 | 1.017 |
| Age | 0.036 | 0.116 | 1.037 | | 0.991 | 1.085 | 0.057 | | 0.002^*^ | 1.058 | 1.021 | 1.097 |
| Gender | 0.183 | 0.632 | 1.201 | | 0.567 | 2.541 | -0.163 | | 0.595 | 0.849 | 0.465 | 1.551 |
| DDM | 0.016 | 0.496 | 1.016 | | 0.970 | 1.064 | 0.009 | | 0.415 | 1.009 | 0.988 | 1.029 |

Notes: ^*^ P<0.05. Abbreviations: DKD, diabetic kidney disease; T2DM, type 2 diabetes mellitus; NAFLD, nonalcoholic fatty liver disease; triglyceride-glucose body mass index, TyG-BMI; triglyceride glucose index, TyG; DDM, duration of diabetes mellitus.
